# Endoscopic Transplantation of Mesenchymal Stem Cell Sheets in Experimental Colitis in Rats

**DOI:** 10.1038/s41598-018-29617-x

**Published:** 2018-07-27

**Authors:** Sehyung Pak, Sung Wook Hwang, In Kyong Shim, Sang Mun Bae, Yeon- Mi Ryu, Han-Byul Kim, Eun-ju Do, Hye-Nam Son, Eun-ji Choi, Sun-ha Park, Sang-Yeob Kim, Sang Hyoung Park, Byong Duk Ye, Suk-Kyun Yang, Nobuo Kanai, Masanori Maeda, Teruo Okano, Dong-Hoon Yang, Jeong-Sik Byeon, Seung-Jae Myung

**Affiliations:** 1 Asan Institute for Life Sciences, Asan Medical Center, University of Ulsan College of Medicine, Seoul, Republic of Korea; 2 Department of Gastroenterology, Asan Medical Center, University of Ulsan College of Medicine, Seoul, Republic of Korea; 3 Research center, Tokyo Metropolitan Geriatric Medical Center, Tokyo, Japan; 4 Institute of Advanced Biomedical Engineering and Science, Tokyo Women’s Medical University, Tokyo, Japan; 5 Cell Sheet Tissue Engineering Center, Department of Pharmaceutics and Pharmaceutical Chemistry, University of Utah, Salt Lake City, Utah USA

## Abstract

Owing to the recent progress in regenerative medicine technology, clinical trials that harnessed the regeneration and immune modulation potentiality of stem cells for treating IBD have shown promising results. We investigated the feasibility and utility of intraluminal endoscopic transplantation of rat MSC sheets in murine models of experimental colitis for targeted delivery of stem cells to lesions. We isolated adipose-derived mesenchymal stem cells (AD-MSC) and bone marrow-derived mesenchymal stem cells (BM-MSC) from EGFP-transgenic rats and fabricated the cells in sheet forms using temperature-responsive culture dishes. The MSC sheets were endoscopically transplanted to the inflamed area in electrocoagulation and DNBS colitis model. The effect of the transplantation was verified using endoscopic scoring and histological analysis. In the electrocoagulation model, the AD-MSC group showed significantly decreased ulcer size in the transplanted regions. In the DNBS colitis model, the AD-MSC group showed decreased inflammation and colitis in the transplanted regions. Histologic analysis showed that the MSC sheets had successfully attached to the inflamed mucosa in both the electrocoagulation and DNBS colitis model. Our results show that endoscopic transplantation of MSC sheets could be a new effective mode of stem cell therapy for IBD treatment.

## Introduction

Inflammatory bowel disease (IBD), which is represented by Crohn’s disease and ulcerative colitis, is characterized by uncontrolled inflammation in gut mucosa due to dysfunctions of innate and acquired immune system^1–3^. Until now, biological agents such as anti-TNF agents have been widely used for the treatment of IBD; however, the therapeutic effect is less than expected^4–6^. Stem cell therapy harnessing the effect of immune regulation ability as well as tissue repairing properties of mesenchymal stem cells (MSCs) has been developed for the treatment of intractable diseases^7^. Among the various kinds of stem cells, MSCs are particularly promising candidates for the treatment of intractable diseases such as perianal fistula in Crohn’s disease due to their capability on regulation of inflammatory response and tissue regeneration^8^. Until now, two methods of MSC delivery have been utilized for IBD treatment—intravenous injection for luminal disease, and local injection for perianal fistula^9–12^. However, intravenous injection method has low survival rate of stem cells in the targeted area in luminal diseases, and thus a very high cell number may be needed to achieve significant therapeutic results. Also, intravenous injection method is prone to producing side effects because the injected stem cells may migrate to undesired sites^7,9^. Therefore, it is reasonable to consider local delivery of MSC for luminal disease, but there is no relevant study on this topic to date.

Recently, injection of colon-specific differentiated organoids has been proposed as an alternative for the intravenous injection method^13,14^. Intrarectal infusion of differentiated organoids from LGR5 (colon stem cell marker)-positive stem cells^14^ isolated from mouse colon resulted in new mucosal formation in the colitis area; however, this method is limited by the need for solidification of materials and the need to temporarily plug the rectum following injection^7,9^. Although organoids have been reported to show high function stability after differentiation from single stem cells under established conditions^15^, stable maintenance of function after transplantation is yet to be confirmed.

To overcome such existing technical limitations, we have tried fabricating MSC cell sheets using a temperature-responsive culture dish, and transplanting them in an endoscopic manner. The cell sheet technology was developed to transfer various types of cells to target organs to be used in various medical fields^16–21^. Even after being removed from the culture dish, the cell sheets retained their extra cellular matrix in an intact form, and they readily attach to the damaged tissue. With such advantage, endoscopic transplantation of cell sheets derived from buccal mucosa was investigated for the prevention of stricture after submucosal dissection of esophagus^21–23^.

According to a recently published literature, direct cell delivery system using cell sheets was shown to be useful for optimized stem cell delivery for treatment of severe heart failure^24^. Focal transplantation to the lesion may be a more practical approach, considering that intravenous injection has a very low rate of peripheral lesion migration and the possibility of being trapped in the lungs when circulating in the blood vessels^25,26^. In this aspect, endoscopic transplantation of MSCs is an appropriate candidate method that allows non-invasive and selective transplantation into the lesions, thereby further enhancing the therapeutic effect of MSCs and avoiding side effects. Reports on the use of cell sheet method in enteric diseases remain scarce; therefore, we aimed to assess the efficacy of endoscopic transplantation of MSCs in two different rat models of colitis. We used electrocoagulation and DNBS colitis model for transplanting MSC sheets by endoscopy, and analyzed the endoscopic phenotypes and histology in the transplanted area.

## Results

### Rat mesenchymal stem cell sheet fabrication

**Figure 1:**
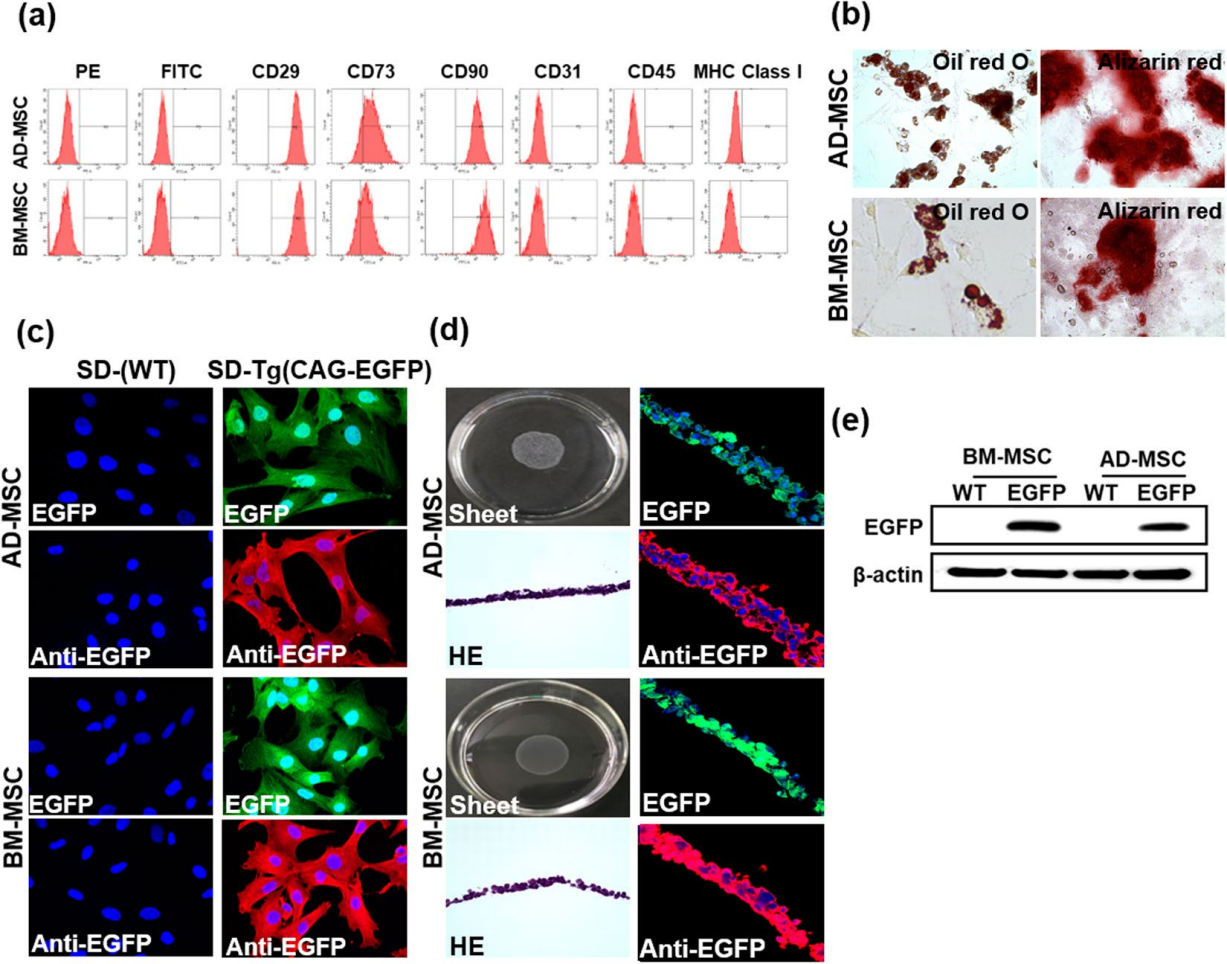
Fabrication of rat mesenchymal stem cell sheets. (**a**) Histogram of cell surface markers showing the AD-MSC (upper) and BM-MSC (lower). (**b**) Differentiation capacity of AD-MSC and BM-MSC for differentiating into adipocytes and osteoblasts with alizarin red. (upper panel; undifferentiated condition, lower panel; differentiated condition). Extracellular calcium deposition in the osteoblasts is shown in red, the accumulation of lipid vacuoles in the adipocytes are shown in red. (**c**) EGFP signal of SD-Tg AD-MSC and BM-MSC 1 day after seeding on a culture dish. AD-MSC and BM-MSC were used at passages 3. (**d**) Fabricated AD-MSC and BM-MSC sheets. AD-MSC and BM-MSC sheets by hematoxylin-eosin staining. EGFP-expressing monolayered AD-MSC and BM-MSC sheets. AD-MSC and BM-MSC were used at passages 3 or 4. (**e**) EGFP protein expression levels in Tg-rat isolated AD-MSC and BM-MSC. All the experiments were performed three times. Green: AD-MSC or BM-MSC from EGFP transgenic rats, Red: EGFP antibody labeled by Alexa Flour 594 Blue: nucleus.

For characterization of mesenchymal stem cells, Adipose-derived mesenchymal stem cells (AD-MSC) and Bone marrow-derived mesenchymal stem cells (BM-MSC) were isolated from SD-rats. Isolated AD-MSC and BM-MSC were stained with cell surface markers and analyzed with flow cytometry using the following markers: hematopoietic stem cell marker; CD45 (negative), endothelial stem cell marker; CD31 (negative), mesenchymal stem cell marker; CD29, CD73, CD90 (positive) (Fig. 1a). In the differentiation analysis, formation of oil and accumulation of calcium in the AD-MSC and BM-MSC was found, showing the differentiation capacity of AD-MSC and BM-MSC (Fig. 1b). For the fabrication of cell sheets, AD-MSC and BM-MSC were isolated from SD(EGFP-CAG)Tg-rats (Tg-Rats). EGFP fluorescence of AD-MSC and BM-MSC isolated from Tg-Rats were used to track the cell sheets after transplantation. EGFP fluorescence through antibody labeling showed an amplified signal than that of the EGFP originated from Tg-Rats (Fig. 1c). Various conditions were applied: (1) Cell numbers; We needed to culture near-confluent amount of cells (8.0 × 10^5^ cells/dish of AD-MSC and 1.1 × 10^6^ cells/dish of BM-MSC) in order to fabricate cell sheets when considering the characteristics of the UPCELL dish. (2) Growth factors; in the UPCELL dish, various growth factors were added to the medium to obtain the best culture condition of the stem cell sheets. The AD-MSC sheets required 20% FBS and 5 ng/ml FGF in culture medium. The BM-MSC sheets required 20% FBS in culture medium. (3) Incubation times; after seeding in the UPCELL dish, the optimal time to detach into cell sheets was measured. As a result, AD-MSC was formed 48 h after cell seeding, and BM-MSC was formed into cell sheets 36 h after seeding as the temperature condition was changed from 37 °C to 25 °C after incubation. After adjusting for the aforementioned conditions, we were able to produce viable cell sheets using AD-MSC and BM-MSC (Fig. 1d). Histological analysis of the AD-MSC and BM-MSC sheets were carried out and it was verified that the sheets were well formed. Immunoblot was performed on EGFP fluorescent tag isolated from Tg-Rats to confirm EGFP expression (Fig. 1e).

### Endoscopic transplantation of MSC sheets in animal models

**Figure 2:**
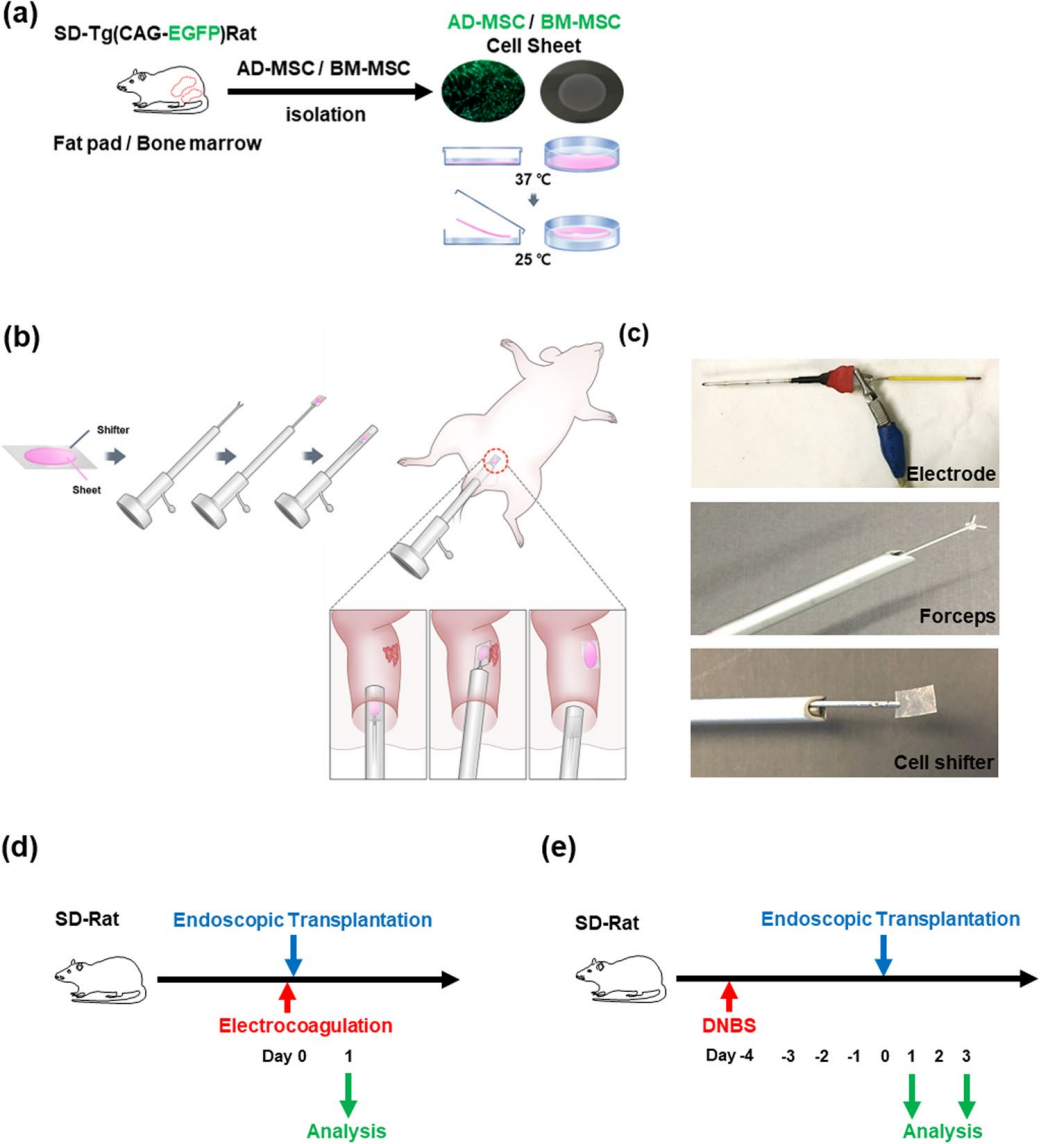
Endoscopic MSC sheets transplantation scheme and animal modeling. (**a**) The fabrication scheme of MSC sheets. (**b**) Endoscopic transplantation process. (**c**) Electrode of electrosurgical equipment for electrocoagulation model (upper). Endoscopic forceps for MSC sheets transplantation (middle). The forceps holding a CELLSHIFTER (lower). (**d**) Study design for electrocoagulation ulcer model. (**e**) Study design for DNBS-colitis model.

A schematic representation of endoscopic transplantation is shown in Fig. 2a,b. AD-MSC and BM-MSC were isolated from Tg-Rats, and were cultured in temperature-responsive culture dishes to fabricate MSC sheets. The fabricated MSC sheets were transferred to the CELLSHIFTER for endoscopic transplantation. The CELLSHIFTER was held with endoscopic forceps and, the MSC sheets was housed in the endoscope sheath (Fig. 2b,c). During the procedure of endoscopic transplantation, the MSC sheets held by forceps were deployed and attached to the transplantation site. The attached area was pressed gently by forceps to reinforce the transplantation of MSC Sheets to the inflamed region. The study schemes of the electrocoagulation ulcer model (Fig. 2d) and DNBS colitis model (Fig. 2e) for the endoscopic transplantation. After electrocoagulation, MSC sheets were transplanted on day 0 and analyzed 1 day after transplantation in electrocoagulation ulcer model. Four days after induction of DNBS colitis, MSC sheets were transplanted and analyzed on days 1 and 3 after transplantation in DNBS colitis model.

### Analysis of electrocoagulation ulcer model after endoscopic transplantation

**Figure 3:**
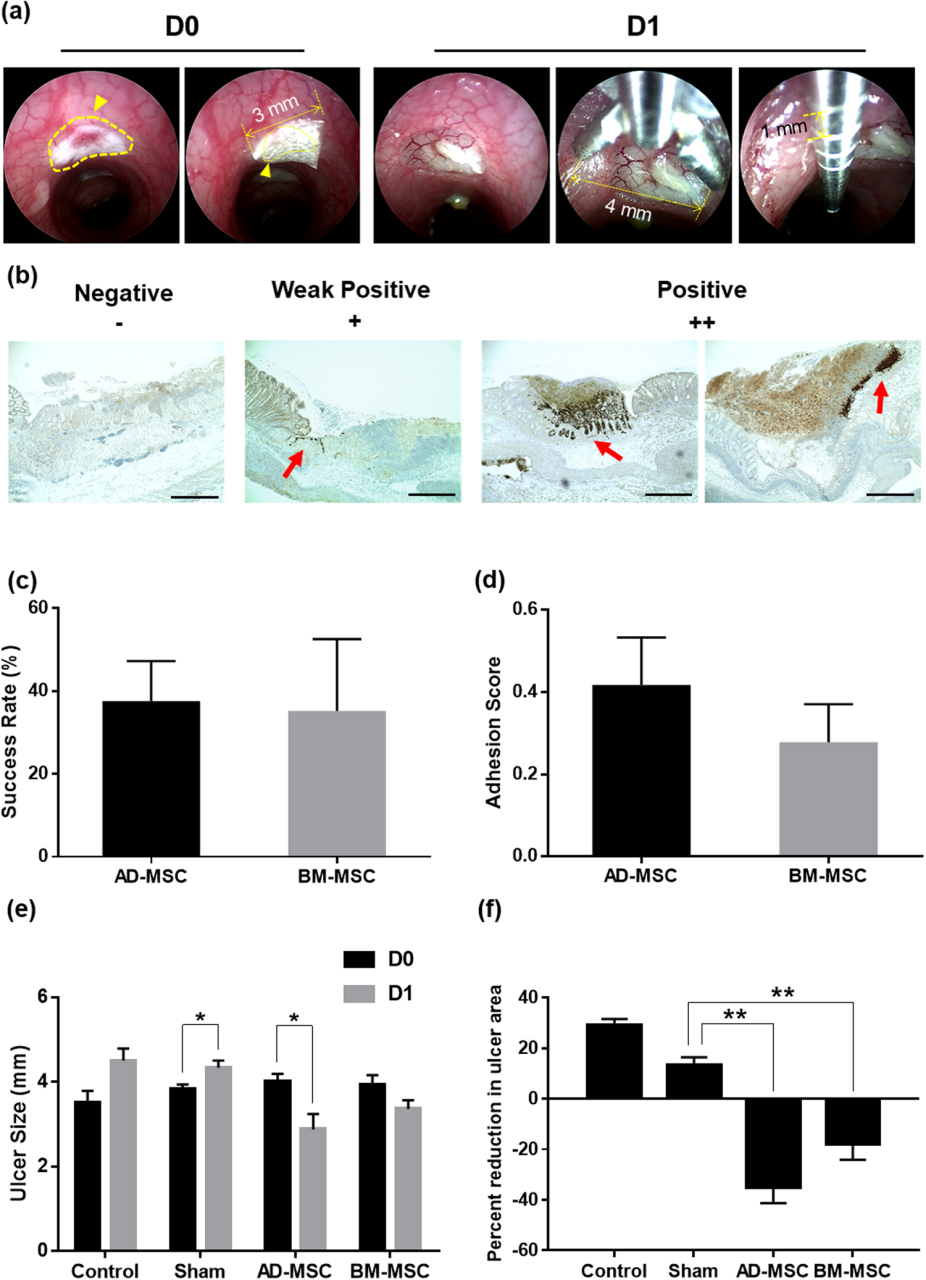
Analysis of the ulcer size change following MSC sheet endoscopic transplantation in electrocoagulation model. (**a**) Cell sheets were transplanted onto the ulcer sites by CELLSHIFTER. The yellow line points to the ulcer site. The yellow arrow indicates the covered MSC sheets by CELLSHIFTER (left panel D0). Evaluation of colonic ulcer size. The maximum opening width of the endoscopic forceps used in the transplantation and the basic length of the forceps wire node (right panel D1). (**b**) Representative histological analysis based on the transplantation success. The red arrow indicates the positive signal of EGFP by immunohistochemistry. (**c**,**d**) Success rate and adhesion score of the electrocoagulation ulcer model. (**e**) The changes in ulcer size before and after transplantation (±SEM n = 8, each). *P < 0.05. (**f**) A comparison of the ulcer size changes after transplantation day (D0) between 1 day after transplant (D1) (±SEM n = 8, each) **P < 0.01. Con; only electrocoagulation, Sham; transplanted without MSC sheets, AD-MSC; AD-MSC sheets transplanted, BM-MSC; BM-MSC sheets transplanted. CELLSHIFTER size: 3 × 6 mm, Forceps width: 4 mm, Forceps wire node length: 1 mm. EGFP positive signal percentage index: negative - 0, week positive - 50, positive – 100. Adhesion score index: negative - 0, week positive - 0.5, positive – 1. MSC sheets transplanted animal, n = 9 (transplanted site, n = 18).

**Figure 4:**
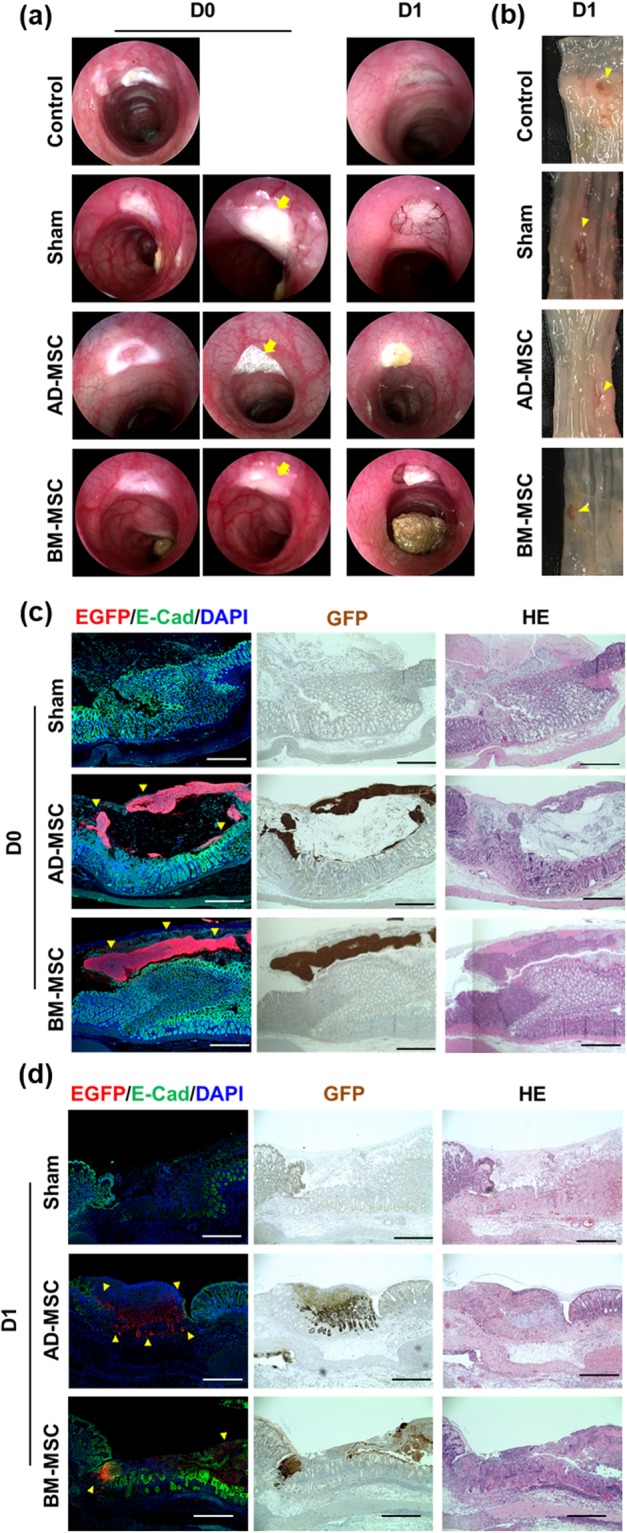
Evaluation of MSC sheet endoscopic transplantation in electrocoagulation model. (**a**) Endoscopic imaging of the colon induced by electrocoagulation ulcer (left). and MSC sheets transplanted at the site of induction of electrocoagulation ulcer (middle). The yellow arrow points to the transplantation site. One day after MSC sheets endoscopic transplantation (right). (**b**) Macroscopic representation of the ulcer formation on colon in 1 day after MSC sheets endoscopic transplantation. The yellow arrow points to the transplanted ulcer site. (**c**) Histological analysis of colon tissue 0 day after MSC sheets. (**d**) Histological analysis of colon tissue 1 day after MSC sheets (middle; AD-MSC, lower; BM-MSC) transplantation group. Con; only electrocoagulation, Sham; transplanted without MSC sheets, AD-MSC; AD-MSC sheets transplanted, BM-MSC; BM-MSC sheets transplanted. The red fluorescent signal is an EGFP signal in immunofluorescence, Dark brown stain was EGFP signal in DAB stain. GFP/E-Cad/DAPI; immunofluorescence stain, Brown GFP; immunohistochemistry, HE; Hematoxylin and eosin stain. Bars represent 200 μm.

Before transplantation, the inflamed colon in each animal was endoscopically observed to select the appropriate sites for MSC sheets transplantation. The following procedure was performed to identify changes in ulcer size before and after transplantation. Cell sheets was transplanted into the ulcer by the CELLSHIFTER. (Fig. 3a left, D0). Evaluation of colonic ulcer size was carried out primarily by using endoscopic forceps. The maximum opening width of the forceps used in the transplantation and the basic length of the wire constituting the forceps were measured in advance and used as references. (Fig. 3a right, D1). The transplanted site was traced with an endoscope on the day of (D0) (Fig. 4a left mid panel) and 1 day after transplantation (D1) (Fig. 4a, right panel). Macroscopic analysis was performed 1 day after MSC sheets transplantation. (Fig. 4b). An endoscopy movie was used to measure the size change of the ulcer occurred from the day of (D0) to the day after transplantation (D1). MSC sheets were transplanted into two sites per animal. We analyzed the success rate for the electrocoagulation ulcer model and performed a histological analysis of the EGFP signal to decide the success of MSC sheet attachment to the damaged lesion site (Fig. 3b). The success rate was 37.60% for AD-MSC group and 35.20% for BM-MSC group (Fig. 3c). The adhesion score was 0.42 for AD-MSC group and 0.28 for BM-MSC group (Fig. 3d). At 1 day after transplantation, the control and sham groups showed an average of 1.00 mm and 0.50 mm increase in diameter, respectively. Conversely, the AD-MSC group showed an average of 1.13 mm decrease in diameter, and the BM-MSC group showed an average of 0.57 mm decrease in ulcer size. In particular, the reduction in ulcer size was significant in the AD-MSC group after one day on the day of transplantation (Fig. 3e). These apparent size changes in ulcer represented 29.17% increase in the control group, 13.10% increase in the sham group, 17.93% reduction in the BM-MSC group, and 35.10% reduction in the AD-MSC group. Consequently, AD-MSC and BM-MSC sheets transplantation effectively reduced the ulcer size compared to the sham group (Fig. 3f). In comparison between AD-MSC and BM-MSC, AD-MSC reduced ulcer size more than did BM-MSC. Colons with and without the transplanted MSC sheets were stained with hematoxylin and eosin or anti-EGFP anti-body, and the transplanted MSC sheets in EGFP-Tg rats were confirmed by immunohistochemistry and immunofluorescence analysis (Fig. 4c,d). Strong EGFP fluorescent signal was observed from the day of the transplantation (Fig. 4c, D0). Analysis of the tissue after one day after transplantation showed that both AD-MSC and BM-MSC cell sheets were retained in the ulcer sites (Fig. 4d, D1). In addition, each MSC sheets migrated to and infiltrated the ulcer area. The patterns of engrafted MSC sheets resembled crypts because they filled the damaged and empty areas.

### Analysis of DNBS colitis model after endoscopic transplantation

**Figure 5:**
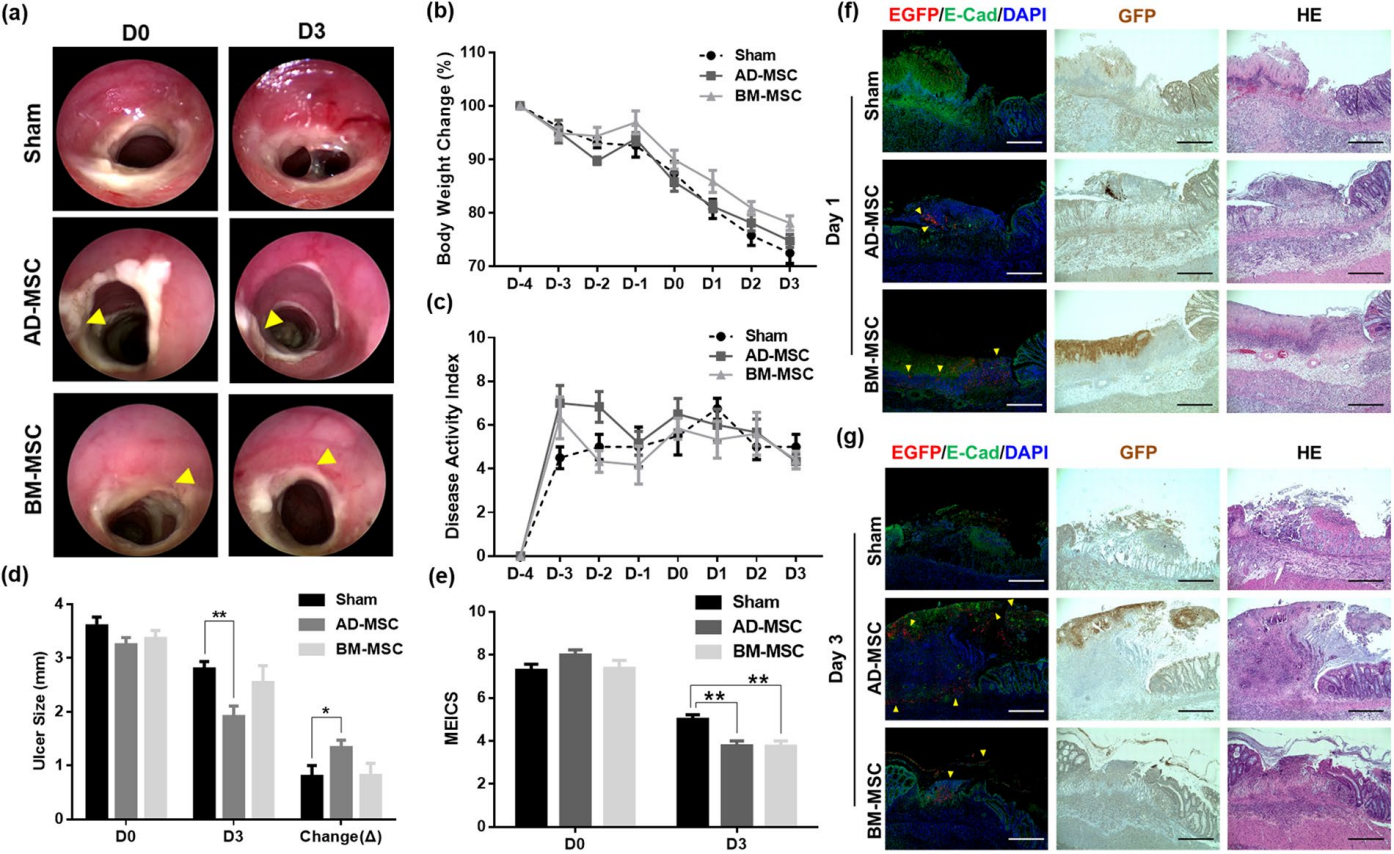
Evaluation of MSC sheets endoscopic transplantation in DNBS colitis model. (**a**) Endoscopic images of MSC sheets transplantation in DNBS colitis model. The yellow arrow points to the transplantation site. (**b**) The time-course body weight change on transplanted and non-transplanted MSC sheets. (**c**) The evaluation of animal model clinical severity assessed by DAI scores. DAI calculated by the combined score of weight loss, stool consistency, and bleeding, as detailed in Table 1. (**d**) The changes in ulcer size before and after D0 between D3 (±SEM n = 12, each). **P < 0.01, *P < 0.05. (**e**) Endoscopic score changes of D0 and D3. D0; transplantation day, D3; 3 days after transplantation (±SEM n = 12, each). **P < 0.01. Sham: transplantation of CELLSHIFTER without MSC sheets, AD-MSC; AD-MSC sheets transplanted, BM-MSC; BM-MSC sheets transplanted. (**f**) Histological analysis of colon tissue 1 day after MSC sheets. (middle; AD-MSC, lower; BM-MSC) attachment group. (**g**) Histological analysis of colon tissue 3 day after MSC sheets. (middle; AD-MSC, lower; BM-MSC) attachment group. Sham; transplanted CELLSHIFTER without MSC sheets, AD-MSC; AD-MSC sheets transplanted, BM-MSC; BM-MSC sheets transplanted. The red fluorescent signal is an EGFP signal in immunofluorescence, Dark brown stain was EGFP signal in DAB stain. GFP/E-Cad/DAPI; immunofluorescence stain, Brown GFP; immunohistochemistry, HE; Hematoxylin and eosin stain. Bars represent 200 μm.

As carried out for the electrocoagulation ulcer model, the transplanted site was traced with an endoscope on the day of (D0) (Fig. 5a left panel) and 3 days (D3) after transplantation (Fig. 5a, right panel). Compared to sham, AD-MSC and BM-MSC groups showed decreases in colitis on endoscopic observation. DNBS colitis was induced 4 days prior to MSC sheets transplantation, and body weight changes (Fig. 5b) as well as disease activity index (DAI) (Fig. 5c) were analyzed following MSC sheets transplantation. There was no significant difference in body weight change and disease activity index among the experimental groups (Fig. 5b,c). In the ulcer size change measurements, sham, AD-MSC, and BM-MSC groups showed reductions of 0.80 mm, 1.33 mm, and 0.82 mm, respectively. The AD-MSC group showed more reduction in ulcer size than did the other groups, the difference between sham and AD-MSC statistically significant (Fig. 5d). According to the MEICS analysis on 3 days after transplantation, the AD-MSC group showed a decrease in its score by 4.22 on average, which was significantly different from that of the sham group. The BM-MSC sheets transplantation group showed a decrease in the average score of 3.63 on D3 compared to D0, which was significantly different from that of the sham group that showed an average score decrease of 2.28 on D3 (Fig. 5e). Histological analysis of DNBS colitis colon confirmed the presence of MSC sheets by fluorescence signal. Hematoxylin and eosin stain, immunohistochemical stain, and immunofluorescence staining with anti-EGFP antibody at 1 day after transplantation of MSC sheets confirmed that the AD-MSC and BM-MSC sheets were attached to the inflammatory site (Fig. 5f). AD-MSC group showed EGFP positive signals in the histological analysis of the colon 3 day after MSC sheets transplantation. The fluorescence intensity was lower than that of day 1. The fluorescence signal of the BM-MSC group was difficult to distinguish from that of day 1 tissues. (Fig. 5g).

## Discussion

To propose a new therapeutic approach in the field of stem cell therapy for IBD, we endoscopically transplanted MSC sheets in rat model colitis and demonstrated the utility of endoscopic transplantation of the MSC sheets. After isolating rat AD-MSC and BM-MSC, we successfully produced AD-MSC and BM-MSC sheets using a temperature-responsive dish. The electrocoagulation model for colonic ulcer was generated with an electrode, and the transplanted site was analyzed to confirm whether the MSC sheets were able to help coagulated ulcers to recover. In the DNBS colitis model, we confirmed the anti-inflammatory effect of MSC sheets transplanted endoscopically onto inflammatory ulcer sites. Our results show that the endoscopic transplantation of MSC sheets onto the inflamed lesions may potentially act as a novel approach for treating IBD.

Until now, cell therapy in IBD treatment has been mainly experimented with hematopoietic stem cell transplantation (HSCT) and MSC transplantation for perianal fistula of Crohn’s dsiease^9,27^. Hematopoietic stem cell transplantation has been studied for a long period, but it can only be applied to a small subset of patients because myeloablation needs to precede the transplantation^9^. In contrast, MSC transplantation has been recognized as a promising therapeutic agent for regenerative medicine in IBD^7^. MSC transplantation is an ideal therapeutic approach because MSC are less immunogenic and easier to extract than other stem cells^28^; therefore, it can be used for treatment after extraction from patients. In addition, MSCs are known to not only regulate immune response, but also promote tissue repair, suggesting further benefits for IBD treatment. Recently, injection of colon-specific differentiated organoids was reported^13,14^; however, this method utilizes solidification materials that are yet to be clinically-approved. In addition, allogenic transplantation of organoids is likely to induce immunogenicity, which may prevent stable delivery and sufficient therapeutic efficacy.

When we compared AD-MSC and BM-MSC cell sheets, the elasticity and viscosity of the cell sheets were higher in AD-MSC sheets. The degree of adherence and histologic analysis of the lesions during transplantation suggested that AD-MSC is superior to BM-MSC. The distinction of paracrine effect between AD-MSC and BM-MSC was not evaluated in this study, but that we will be compared in further studies. The paracrine effect on adjacent tissues was greater in AD-MSC than in BM-MSC^29^. According to the reference analyzing differences between different types of MSCs, it is supported that AD-MSC share an immunosuppressive property like BM-MSC. Thus, AD-MSC-based regeneration therapy can use allogeneic cells. Considering the MSC sampling procedure for autologous transplantation from patients, AD-MSC may be a good alternative to BM-MSC^30,31^.

The electrocoagulation ulcer model was used to recapitulate wound healing scenario and to confirm the effects of cell sheet transplantation on ulcer size reduction. The ulcer size showed a significant reduction on the MSC sheets transplantation condition. In the DNBS colitis model, the ulcer size and MEICS of lesion were significant changed, and there was no significant difference in body weight and DAI-expressing overall inflammation. We used two type of cell sheets for transplantation. Based on the results of our DNBS colitis model, a suitable direction for further study would be to cover as many colitis lesions as possible via multiple grafting at one- or two-day intervals. Cell sheets do not ameliorate overall inflammation, but they can be effectively applied to local sites with severe pathologies. The fistula model may be appropriate for use in further studies.

The limitations of this study include the lack of long-term observation following MSC sheets transplantation, which was because both the electrocoagulation model and the DNBS colitis model rats recovered within 4 to 5 days of the experiment, thus making it difficult to observe and compare the long-term effects of the disease and therapeutic maneuvers. Also, the scope of the current study did not include investigation of the molecular mechanisms and the immune response analysis on the transplanted area.

Despite these limitations, we have clearly shown that MSC sheets reduces ulcer induced from physical mucosal damage (electrocoagulation model), and mitigates the severity of inflammation due to colitis condition (DNBS model); therefore, therapeutic effect on IBD through modulation of immune response may also be expected. Also, no beneficial effects were observed in body weight change and disease activity index in DNBS colitis model because the endoscopic transplantation of MSC sheets was not enough to cover the entire colon ulcer in colitis model. Therefore, we suggest that our method be applied as an adjunctive method to the existing IBD treatment by first applying to serious colitis sites^32,33^.

From the results presented, it is worthwhile to note that there was significant reduction in ulcer size and colitis score for AD-MSC and BM-MSC sheets transplantation in both electrocoagulation and DNBS model. The benefits of our endoscopic transplantation of MSC sheets seem to be largely due the direct application of cells onto the lesions. This novel method described herein has no need for scaffolds or solidification of cells to enhance cell attachment, and the MSCs themselves retain the characteristics of ECM. Our results suggest that MSC sheets is a highly practical technology for clinical application especially for enteric diseases, because cell sheets can be directly applied to colonic lesions via endoscope to exert immediate protective effect. In addition, endoscopic transplantation is safer than conventional single cell injection technique and has a high potential for stable transplantation into the lesion sites. MSC sheets from can be directly transplanted with endoscopes, making them suitable for treating various symptoms of colon disease and restoring tissue integrity of the surgical site. In conclusion, our results show that endoscopic transplantation of MSC sheets could be a new effective stem cell therapy for treatment of IBD.

## Materials and Methods

### Isolation and characterization of rat mesenchymal stem cells

AD-MSC were isolated from the inguinal adipose tissue of SD(CAG-EGFP) transgenic rats, which was processed according to a previously reported method^34,35^. Briefly, the isolated adipose tissue was enzymatically digested. The stromal vascular fraction was collected after centrifugation at 700 × g for 5 min. Cells in the stromal vascular fraction were plated on tissue culture dish and cultured in complete culture medium consisting of DMEM/F-12 (Invitrogen) with 10% FBS (Hyclone, Thermo Scientific, Landsmeer, The Netherlands) and 1% Antibiotic-Antimycotic (A-A) (Invitrogen) at 37 °C in a 5% CO_2_ incubator. After 24 h, debris was removed by washing with PBS (Life Technologies, Grand Island, NY, USA), and fresh complete culture medium was added. The cells were passaged on day 2 and transferred to a new dish. Subcultures were plated at a density of 2.0 × 10^5^ cells/10-cm^2^ every 3 days. BM-MSC were isolated from bilateral femurs and tibias of Tg-Rats, which was processed according to a previously reported method^36,37^. A single suspension of bone marrow-derived all nuclear cells was seeded in tissue culture dish and incubated at 37 °C with 5% CO_2_ incubator. After 12 h, nonadherent cells were removed, and adherent cells were cultured in complete culture medium consisting of DMEM + GlutaMAX (Invitrogen) supplemented with 10% FBS and 1% A-A. The medium was replaced and washed every day while growing the cells. The adherent cells were passaged with frequent medium changes to eliminate potential hematopoietic cell contamination.

AD-MSC and BM-MSC at passage 3 were suspended and incubated with an PBS containing 2% FBS, followed by primary antibodies: CD31 (#555027), CD45 (#554878), CD73 (#551123), CD29 (#562154) (BD biosciences), CD90 (Ab226), CD105 (Ab11414) (Abcam, Cambridge, UK). The cells were analyzed with a flow cytometer (CANTO II; BD biosciences).

AD-MSC and BM-MSC were confirmed by measuring their adipogenic and osteogenic abilities, respectively, by using previously reported methods^38^. For each assay, AD-MSC and BM-MSC at passage 3 were plated in a 6-well plate (Nunc, Roskilde, Denmark) and cultured in complete culture medium. For adipogenesis, the medium was switched to the STEMPRO Adipogenesis Differentiation medium (Life Technologies). After 14 days, the cells were fixed with 4% paraformaldehyde phosphate buffer solution (PFA) (Wako, Osaka, Japan) for at least 1 h and stained for 2 h with fresh Oil Red-O solution (Wako, Osaka, Japan). For osteogenesis, the medium was switched to the STEMPRO Osteogenesis Differentiation Medium (Life Technologies). The cells were incubated for 30 days and then stained with 1% alizarin red-S solution. Each experiment was performed in triplicates.

### Fabrication of rat mesenchymal stem sheets using temperature-responsive culture dish

AD-MSC and BM-MSC at passage 3–4 were seeded on 35-mm diameter temperature-responsive culture dishes, UPCELL (CellSeed, Tokyo, Japan). The UPCELL dish was pre-coated with 2 ml FBS for 30 min in an incubator before seeding the stem cells. AD-MSC were seeded at 8.0 × 10^5^ cells/dish and cultured in DMEM/F-12 medium with 20% FBS, 5 ng/ml FGF (Invitrogen) and 1% A-A for 48 h. BM-MSC were seeded at 1.1 × 10^6^ cells/dish and cultured in DMEM + GlutaMAX medium supplemented with 20% FBS and 1% A-A for 36 h. After reducing the temperature to 25 °C in a CO_2_ incubator, the cells spontaneously detached as contiguous cell sheets and were harvested from the dishes with the supplied membrane CELLSHIFTER (CellSeed, Tokyo, Japan).

### Animals

Specific pathogen free six weeks-old (150–200 g) male Sprague Dawley (SD) rats (Orient Bio Inc., Seongnam, Korea) and SD(EGFP-CAG)Tg rats (SLC, Tokyo, Japan) were used. All animal experiments were performed in accordance with protocols approved by the Institutional Animal Care and Use Committee (IACUC) of the Asan Institute for Life Sciences at Asan Medical Center, consistent with the Institute of Laboratory Animal Resources (ILAR) guidelines. All experiments were performed in accordance with relevant guidelines and regulations. The animals were housed in standard laboratory conditions with a temperature of 21–23 °C and 12 h dark/light cycles. Rats were allowed *ad libitum* access to food and water throughout the study period.

### Chemicals and devices

2,4-Dinitrobenzene sulfonic acid dihydrate (DNBS) was purchased from MP Biomedicals (Aurora, OH, USA). Endoscopic transplantation was carried out using Karl Storz COLOVIEW mini-endoscopic system (KARL STORZ GmbH & Co. KG, Tuttlingen, Germany). For electrocoagulation model, ZATHA Electrosurgical Unit (Jejoong medical, wonju, korea) was used.

### Electrocoagulation model and DNBS colitis model

Electrocoagulation model was generated according to a previously reported method^39,40^. For experimental purposes, animals were fasted for 12 h with *ad libitum* access to 8% sucrose water in 0.2% saline to prevent dehydration. The 10-cm electrocautery probe (electrode) was made of platinum-coated copper node. The electrode was insulated except for both extremities. For electrocoagulation, the ball-shaped tip of the electrode was introduced through the anus into the 5 cm and 3 cm depths of colon and directly contacted the mucosa; the other tip was connected to the pole of a direct current generator. The position of the tip of electrode directly contacting the mucosa was determined by the direction of the experimenter’s finger from the lower abdomen. Bipolar current (18~20 W) was delivered through the electrodes for 2 sec. During endoscopic observation, the ulcer diameter was measured using the biopsy forceps (maximum opening width: 4 mm). Induction of DNBS colitis model was based on the previously reported protocols^41,42^. Briefly, 30 mg/250 μl (in 50% EtOH) DNBS was intrarectally infused via a polyethylene (PE) zonde (Φ 1.8 × 95 mm) into 6 weeks old SD rats under isoflurane-induced anesthesia.

### Endoscopic transplantation of MSC sheets

The experimental groups of the electrocoagulation model were as follows: Control; no procedures performed after electrocoagulation induction, Sham; transplantation with a CELLSHIFTER without MSC sheets after electrocoagulation, AD-MSC, BM-MSC; electrocoagulation followed by transplantation of AD-MSC or BM-MSC sheets. Endoscope was used to confirm the location and size of the ulcer and to transplant the MSC sheets onto the electrocautery-induced ulcers. The experimental groups of the DNBS colitis model were as follows: Normal; simulated transplantation only with a CELLSHIFTER without MSC sheet in healthy condition, Sham (DNBS control); induced DNBS colitis model, transplantation with a CELLSHIFTER without MSC sheets, AD-MSC, BM-MSC; induced DNBS colitis model, followed by transplantation of AD-MSC or BM-MSC sheets. The MSC sheets were transferred to the CELLSHIFTER that was included with the UPCELL dish using a pair of forceps specifically designed for the endoscope. Then, the MSC sheets laid onto the forceps was attached to the target area. The site of transplant was selected as the inflammatory site adjacent to the mucus from the colitis in DNBS colitis model. This endoscopic transplantation of DNBS colitis model was performed on stool- and mucus-removed areas where the cell sheet was physically attached to.

After transplantation, we utilized an endoscope to accurately locate and analyze the transplanted area. On the day of transplantation and 1 day after transplantation in the EC model, the rats were sacrificed for macroscopic observation and fixation for histologic analysis. (EGFP positive signal percentage index: negative - 0, week positive - 50, positive - 100. Success rate score index: negative - 0, week positive - 0.5, positive - 1) (MSC cell sheet transplanted animal, n = 9) In the DNBS colitis experiments, the rats were sacrificed at either 1 day or 3 days post-transplantation for histologic analysis. The evaluation of colonic ulcer size was carried out primarily by using endoscopic forceps. The maximum opening width of the forceps used in the transplantation and the basic length of the wire constituting the forceps were measured in advance and used as references. A complementary approach was to use the CELLSHIFTER for transferring cell sheets during the endoscopic transplantation. The size of CELLSHIFTER was 3 × 6 mm. It was used as a scale in the endoscopic evaluation after being applied in uniform size.

### Assessment of disease activity in DNBS colitis model

The disease activity of the DNBS models was evaluated by the sum of the scores in each category—a percentage of weight loss from body weight, characteristics of the stool and the presence of blood—from the day of DNBS administration to the day of sacrifice following MSC sheets transplantation (Supplementary Table 1)^43,44^. The endoscopic score DNBS colitis severity was evaluated by the sum of the scores in murine endoscopic index of colitis severity (MEICS)—thickening of the colon, changes of the vascular pattern, fibrin visible, granularity of the mucosal surface and stool consistency—from the day of DNBS administration to the day of sacrifice following MSC sheets transplantation (Supplementary Table 2)^43,44^.

### Histological analysis of the colon tissue

At 1 h after transplantation and the day after, the lower abdomen of the model was excised and the colon was removed. The colon was longitudinally sectioned to confirm the attachment of the MSC sheets on the target area. The subsequent process followed the aforementioned reference^45^. Tissue sections were incubated consecutively overnight at 4 °C with primary antibodies, rabbit anti-GFP (MBL, #598; 1:200, Nagoya, Japan) and FITC mouse anti-E-Cadherin (BD bioscience, #612131; 1:300). Then the sections were incubated for 1 h at room temperature with Alexa Fluor 594-conjugated goat anti-rabbit secondary antibodies (Cell signaling, #8889, 1:500; MA, USA). Serial sections of tissue blocks were subjected to either hematoxylin-eosin staining and immunohistochemical staining. Tissue sections were incubated overnight at 4 °C with primary antibodies, rabbit anti-GFP (MBL, #598; 1:2000). The sections were counterstained with Harris hematoxylin. The extent of the MSC sheets attachment and changes in the surroundings of the transplanted area were analyzed according to a previously published protocol^41^. Immunofluorescence stained tissues were visualized by confocal scanning microscopes (LSM 780; Carl Zeiss Microscopy GmbH, Germany). Bright-field images (HE and immunohistochemical stain) were obtained by auto imaging system (EVOS FL Auto; Life Technologies).

### Statistical analysis

Quantitative data are presented as means ± SEM and were analyzed with nonparametric test, Mann-Whitney test. Statistical analysis was performed with IBM SPSS 21.0 software (Armonk, NY, USA). P-values less than 0.05 were considered statistically significant. All experiments were performed at least three times per group and at least three experiments were performed. Tissue staining results are representative of at least three independent determinations. The number of samples are indicated in the figure legends.

## Electronic supplementary material

Supplementary information
