# Supplementary material for: Endoscopic Transplantation of Mesenchymal Stem Cell Sheets in Experimental Colitis in Rats
Source: Sci Rep. 2018 Jul 27;8:11314. doi: 10.1038/s41598-018-29617-x (PMC6063883; doi:10.1038/s41598-018-29617-x)
Supplement: Supplementary file 1 — Supplementary information [file 41598_2018_29617_MOESM1_ESM.docx]

**Endoscopic Transplantation of Mesenchymal Stem Cell Sheets in Experimental Colitis in Rats**

**Sehyung Pak, Sung Wook Hwang, In Kyong Shim, Sang Mun Bae, Yeon- Mi Ryu, Han-Byul Kim, Eun-ju Do, Hye-Nam Son, Eun-ji Choi, Sun-ha Park, Sang-Yeob Kim, Sang Hyoung Park, Byong Duk Ye, Suk-Kyun Yang, Nobuo Kanai, Msanori Maeda, Teruo Okano, Dong Hoon Yang, Jeong-Sik Byeon and Seung-Jae Myung**

**Supplementary table**

Table 1. Scoring system for calculating disease activity index (DAI)

| **score** | **weight loss** | **Stool consistency** | **Occult/gross bleeding** |
| --- | --- | --- | --- |
| **0** | none | normal | normal |
| **1** | 1-5% | - | - |
| **2** | 5-10% | loose stools | slight bleeding |
| **3** | 10-15% | - | - |
| **4** | >15% | diarrhea | gross bleeding |

Table 2. Endoscopic colitis score (murine endoscopic index of colitis severity; MEICS)

|  | Murine endoscopic index of colitis severity (MEICS) | | | |  |  |
| --- | --- | --- | --- | --- | --- | --- |
|  |  |  |  |  |  |  |
|  | 0 | 1 | 2 | 3 |  | Total |
| Thickening of the colon | Transparent | Moderate | Marked | Non-transparent |  | 0–3 |
| Changes of the vascular pattern | Normal | Moderate | Marked | Bleeding |  | 0–3 |
| Fibrin visible | None | Little | Marked | Extreme |  | 0–3 |
| Granularity of the mucosal surface | None | Moderate | Marked | Extreme |  | 0–3 |
| Stool consistency | None + Solid | Still shaped | Unshaped | Spred |  | 0–3 |
|  |  |  |  |  |  | Overall: 0–15 |

**Supplementary figures**


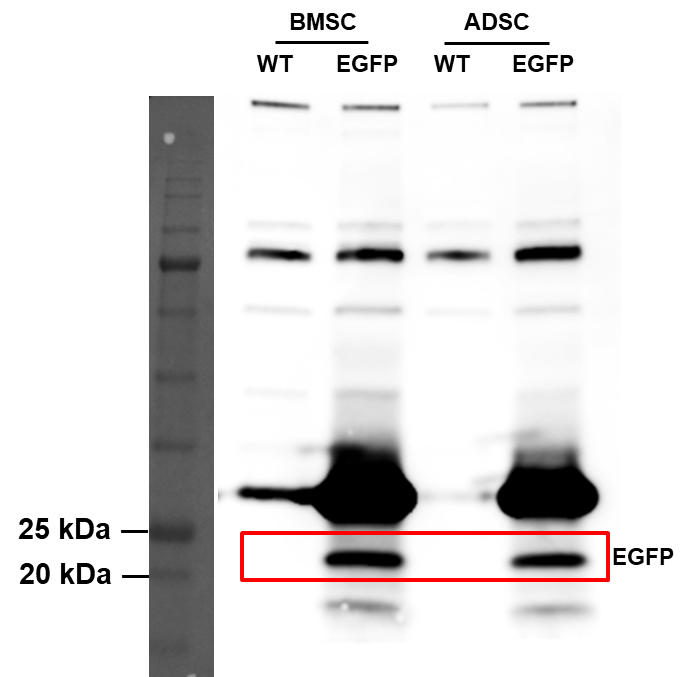


**Figure 1. Fabrication of rat mesenchymal stem cell sheets.** (**e**) full-length blots of EGFP protein expression. EGFP protein expression levels in Tg-rat isolated ADSCs and BMSCs.


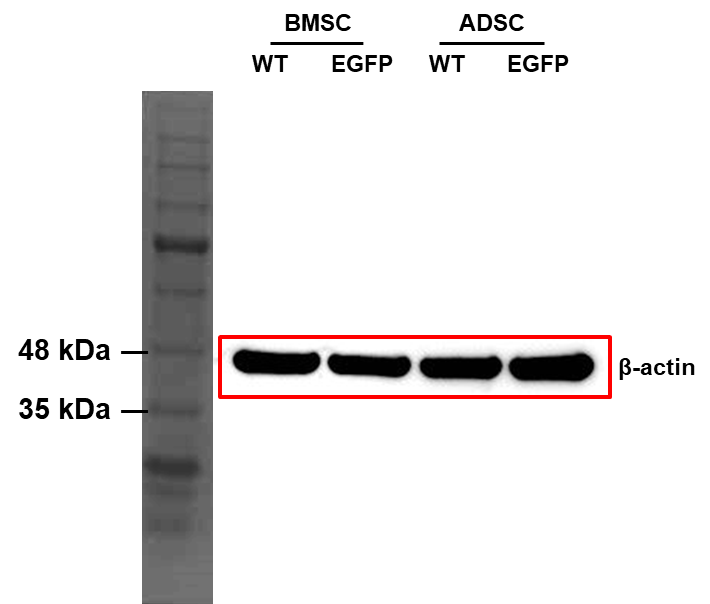


**Figure 1. Fabrication of rat mesenchymal stem cell sheets.** (**e**) full-length blots of beta-actin protein expression for loading control. EGFP protein expression levels in Tg-rat isolated ADSCs and BMSCs.
